# Cost-Effectiveness Analysis of Older Adult Vaccination with the Bivalent Respiratory Syncytial Virus Prefusion F (RSVpreF) Vaccine in Mexico

**DOI:** 10.3390/vaccines14080713

**Published:** 2026-08-19

**Authors:** Veronica Guajardo, Ali Shajarizadeh, Nishu Gaind, Luka Ivkovic, Rengina Kefalogianni, Diana Mendes

**Affiliations:** 1 Pfizer, Mexico City 05120, Mexico; 2 Evidinno Outcomes Research Inc., Vancouver, BC V5Y 1K2, Canada; ashajarizadeh@evidinno.com (A.S.); nnishu@evidinno.com (N.G.); livkovic@evidinno.com (L.I.); 3 Pfizer Ltd., Tadworth, Surrey KT20 7NS, UK; rengina.kefalogianni@pfizer.com (R.K.); diana.mendes@pfizer.com (D.M.)

**Keywords:** respiratory syncytial virus (RSV), older adults, Mexico, RSVpreF, vaccination program, cost-effectiveness, economic evaluation, Markov model, healthcare system perspective, societal perspective

## Abstract

Background/Objectives: Respiratory syncytial virus (RSV) causes substantial morbidity and mortality in older adults, and in Mexico’s rapidly growing older-adult population it may place increasing pressure on hospital-based care; however, Mexico-specific evidence to inform adult RSV immunization policy remains limited. This study estimated the long-term clinical and economic burden of medically attended RSV among adults aged 60–99 years in Mexico and evaluated the health impact and cost-effectiveness of a year-round RSVpreF vaccination program. Methods: A population-based Markov cohort model compared the RSVpreF vaccination with no vaccination in a hypothetical Mexican cohort aged 60–99 years over a lifetime horizon. Outcomes included RSV-related hospitalizations, emergency department (ED) and physician office (PO) encounters, in-hospital deaths, life-years (LYs), and quality-adjusted life-years (QALYs). Analyses were conducted from Mexican healthcare system and societal perspectives in 2025 Mexican pesos (MXN$) and US dollars (US$), with costs and outcomes discounted at 5% annually. One-way and probabilistic sensitivity analyses and scenario analyses assessed the robustness of the findings. Results: With 58% uptake, RSVpreF reduced hospitalizations by 187,825, ED encounters by 178,278, PO encounters by 465,976, and RSV-related deaths by 15,384. In the first 5 years, hospitalizations, ED encounters, and deaths declined by 31% each, and PO encounters by 14%. Over the lifetime horizon, vaccination generated an additional 96,227 discounted LYs and 71,526 discounted QALYs, while avoiding MXN$ 19,484 million (US$ 1061 million) in direct medical costs and MXN$ 3324 million (US$ 181 million) in indirect costs. Conclusions: Year-round RSVpreF vaccination in Mexico among adults aged 60–99 years could substantially reduce medically attended RSV cases and RSV-related mortality and is projected to be cost-effective, thereby supporting the adoption of preventive strategies to address the growing clinical and economic burden of RSV in Mexico’s aging population.

## 1. Introduction

Respiratory syncytial virus (RSV) is a common respiratory pathogen that typically causes mild upper respiratory symptoms but can progress to severe lower respiratory tract infection (LRTI), including bronchiolitis and pneumonia, particularly in vulnerable groups such as older adults and individuals with underlying medical conditions. Advanced age and chronic comorbidities (e.g., cardiopulmonary disease, diabetes, immunocompromising conditions) have been consistently associated with an increased risk of RSV-related complications, hospitalization, and death [1]. Although much of the epidemiologic evidence in adults comes from high-income countries, these risk patterns are expected to be at least as pronounced in middle-income settings, where healthcare access and capacity constraints may further worsen outcomes [1]. In middle-income settings with constrained healthcare capacity, preventable RSV-related hospitalizations among older adults may place disproportionate pressure on health systems, magnifying both the clinical and economic consequences.

Evidence from Latin America and the Caribbean (LAC) indicates that RSV is an important contributor to severe respiratory disease and healthcare utilization across the life course, including among older adults and individuals with comorbidities. A recent systematic review and meta-analysis compiling data from LAC countries reported a meaningful RSV-associated burden in both children and adults, and highlighted variability in reported outcome estimates across settings, reflecting differences in surveillance, healthcare access, and population risk profiles [2]. Together, these findings reinforce that adult RSV burden is relevant in the region and underscore the need for robust, country-specific evidence to inform policy decisions in settings such as Mexico.

Mexico is undergoing rapid demographic aging. According to the National Population Council (CONAPO), approximately 18.3 million adults aged 60–99 years were living in Mexico in 2025, representing 13.8% of the total population, up from 10.9% in 2015, and projected to reach 17.8% by 2035 [3]. As this older population grows, demand for hospital-based respiratory care and associated healthcare expenditures are expected to increase, strengthening the rationale for adopting preventive strategies in older adults. Many of these older adults have one or more chronic comorbidities; a large national COVID-19 cohort study showed that age and multimorbidity were key predictors of severe respiratory outcomes in Mexico, underscoring the clinical vulnerability of this group to respiratory pathogens such as RSV [4].

Although evidence continues to build, quantifying the RSV disease burden in adults is hindered by substantial under-ascertainment, as routine RSV testing is uncommon and symptoms are non-specific and overlap with other respiratory infections, leading to misclassification in administrative and surveillance data [5,6,7]. These challenges are amplified in LAC, including Mexico, where gaps in surveillance and diagnostic practices contribute to under-recognition of RSV burden in older adults [6]. Using national hospitalization data within the Global Burden of Disease framework, Burkart et al. report adjusted RSV-attributable cardiorespiratory hospitalization incidence estimates for Mexico, with an incidence of 533 per 100,000 person-years among adults aged ≥60 years [8]. Case-fatality rates (CFRs) from a systematic review of RSV in older and high-risk adults suggest that around 8–10 deaths may occur per 100 RSV-related hospitalizations in this age group, with mortality increasing further at older ages [9]. Together, these figures highlight RSV as an important cause of severe respiratory disease and death among older adults in Mexico, particularly those with underlying comorbidities.

The economic consequences of RSV in Mexican older adults are also considerable. In the context of structural budget constraints, RSV hospitalizations may represent an under-recognized driver of healthcare spending and create opportunity costs by displacing resources from other priority conditions. Claims-based analyses from other settings have similarly shown high healthcare utilization and costs associated with RSV in adults, during and beyond the acute disease stage, reinforcing its economic relevance as a vaccine-preventable disease [10,11,12,13,14].

Until recently, preventive options for RSV in older adults in Mexico were limited to general infection-control measures; no RSV vaccine was available for this age group. In 2024, the Federal Commission for Protection against Health Risks (COFEPRIS) authorized an RSV prefusion F (RSVpreF) vaccine for use in pregnant women and adults aged ≥60 years, creating a new opportunity to reduce RSV burden across the life course in Mexico [15]. Maternal RSVpreF vaccination during pregnancy provides passive protection to infants through transplacental transfer of antibodies, thereby indirectly protecting this vulnerable group. The bivalent RSVpreF vaccine demonstrated high efficacy against RSV-associated LRTI in the phase 3 (RSV Vaccine Efficacy Study in Older Adults Immunized Against RSV Disease [RENOIR]) clinical trial in older adults [16] and early real-world data from Kaiser Permanente Southern California have reported substantial effectiveness against RSV-related hospitalizations and emergency department (ED) visits over the first two post-licensure seasons [17,18]. Mexico also has existing infrastructure for adult respiratory vaccination: data from the 2023 National Health and Nutrition Survey (ENSANUT) indicate that influenza vaccination coverage among adults aged ≥60 years reached approximately 58%, providing a realistic benchmark for potential RSV vaccine uptake in older adults [19]. Together, the availability of an effective vaccine and established adult immunization infrastructure presents a timely opportunity to shift from reactive management of severe RSV cases toward a more preventive, value-oriented strategy.

The current global RSV vaccine landscape includes three vaccines authorized for use in older adults in multiple jurisdictions: the bivalent protein-based RSVpreF vaccine, the adjuvanted protein-based RSVPreF3 vaccine, and the mRNA-1345 vaccine [20]. The bivalent RSVpreF vaccine demonstrated high efficacy against RSV-associated lower respiratory tract illness in the Phase 3 RENOIR randomized controlled trial, with subsequent real-world evidence confirming its effectiveness against RSV-related hospitalizations and emergency department visits [16,17,18]. Among the available vaccines, RSVpreF is the only bivalent vaccine and is also authorized for maternal immunization to protect infants during the first months of life [20]. Recommendations for adult RSV vaccination differ across countries according to age, clinical risk, implementation strategy, and funding arrangements. In the United States, a single dose is recommended for all adults aged ≥75 years and adults aged 50–74 years at increased risk of severe RSV disease [21]. In Canada, publicly funded immunization programs are recommended for all adults aged ≥75 years and adults aged 65–74 years at increased risk of severe RSV disease [20]. European recommendations are heterogeneous but generally prioritize adults aged ≥75 years and younger adults with risk factors [22]. In Latin America, Panama introduced RSV vaccination through its public immunization program for adults aged ≥60 years in 2025 [23]. This evolving international policy landscape underscores the importance of generating country-specific evidence to inform vaccination eligibility, implementation, and funding decisions in Mexico.

In Mexico, decisions to introduce new adult vaccines into national programs must be informed by robust local evidence on both health impact and economic value. To date, however, there has been no comprehensive evaluation of the long-term clinical and economic consequences of RSV among older adults in Mexico, nor of the cost-effectiveness of RSVpreF vaccination in this population. This study therefore aimed to estimate the clinical and economic burden of RSV-associated LRTI among adults aged 60–99 years in Mexico and to evaluate the health impact, cost-effectiveness, and value-based price of implementing a year-round RSVpreF vaccination program in this population from healthcare system and societal perspectives.

## 2. Materials and Methods

### 2.1. Model Overview

An existing, previously peer-reviewed, population-based Markov cohort model developed in Microsoft Excel for Microsoft 365 (Microsoft Corporation, Redmond, WA, USA) was adapted [24,25,26] to project the clinical and economic outcomes of RSV over a lifetime horizon and the impact of single-dose RSVpreF vaccination among older adults in Mexico (Figure S1). A cohort-based Markov framework was selected because it is well suited to projecting long-term outcomes in an aging population by tracking time-varying risks, recurrent events, costs, and quality of life over a lifetime horizon in a transparent and reproducible structure. The model simulates a hypothetical cohort of adults aged 60–99 years, structured in 1-year age bands and stratified by RSV risk status. At model entry, the cohort reflects the age and risk distribution of the Mexican older adult population, and individuals are then followed in monthly cycles until death or a maximum age of 99 years. While vaccine protection was assumed to decline linearly and reach 0% after 90 months, the clinical benefits accrued during the period of protection—such as prevention of premature deaths—may continue to affect long-term survival and quality-adjusted life years (QALYs). These downstream effects justify the use of a lifetime horizon.

Besides the extensive quality checks for internal/external validation, the economic model was also subject to thorough evaluation by an internal senior health economist for this adaptation, which included internal model validation and ensured that the selected modeling approaches were methodologically sound.

Within each cycle, the model applies a Markov-type state-transition process to estimate RSV-related health events and associated costs as a function of age, risk group, vaccination status, and time since vaccination. Clinical outcomes include medically attended RSV cases stratified by care setting: hospitalizations, ED visits, and physician office (PO) visits, as well as RSV-associated in-hospital deaths, life-years (LYs), and quality-adjusted life-years (QALYs). Economic outcomes comprise vaccination program costs, direct medical costs of RSV care, and indirect costs related to morbidity- and mortality-associated productivity losses among older adults and their caregivers.

### 2.2. Population

For this analysis, the entire population of Mexican older adults aged 60–99 years was considered. According to the National Population Council (CONAPO) projections for 2025, there are approximately 18.3 million individuals in this age range in Mexico, accounting for about 13.8% of the country’s total population [3]. These 18,347,430 seniors formed the basis of our modeled population. In the model, adults aged 60–99 years were represented in four age strata (60–64, 65–74, 75–84, and 85–99).

This older adult cohort was stratified into risk categories based on their underlying health status. The number of individuals at each risk level was estimated using proportions derived from a large national COVID-19 cohort study in Mexico by Domínguez-Ramírez et al. (2023) [4]. This study reported that the majority of severe COVID-19 outcomes in older adults occurred in those with at least one chronic comorbidity (such as diabetes, hypertension, or obesity), with various combinations of these conditions accounting for roughly 77–84% of COVID-19 deaths [4]. Accordingly, our model divided the 60–99-year population into a high-risk group (individuals with one or more of these risk-enhancing comorbidities) and a low-risk group (individuals without such conditions), using the risk level distribution reported in that cohort study to allocate individuals to each group (Table S1). This approach ensured that the simulated population’s risk profile reflects the observed epidemiological pattern in Mexico’s older adults during the COVID-19 pandemic. The comorbidity-based risk stratification was considered as a proxy for RSV risk because of the presence of common chronic conditions (e.g., cardiopulmonary disease, diabetes, obesity) that are well-established predictors of severe RSV outcomes in older adults. In the absence of RSV-specific data, the comorbidity distribution from this large, nationally representative cohort provides a pragmatic and epidemiologically relevant basis for distinguishing higher- versus lower-risk subgroups for RSV in the older adult population.

### 2.3. Epidemiology

The model included medically attended RSV cases in three settings: hospitalizations, ED visits, and PO visits.

For hospitalizations, Mexico-specific RSV-attributable cardiorespiratory hospitalization rates from Burkart et al., who estimated age-specific RSV hospitalizations across several countries, including Mexico, were used [8]. These age-based rates were converted to the model’s age bands.

For RSV ED and PO visits, age-specific outpatient RSV incidence from Li et al. [27] was used and mapped to the model’s age bands.

To derive risk-stratified incidence estimates across all three settings (hospitalizations, ED visits, and PO visits), relative risks (or rate ratios) by comorbidity profile from Weycker et al. [28] together with the age-specific proportions of high- and low-risk adults derived from the Mexican cohort study by Domínguez-Ramírez et al. [4] was applied, disaggregating the total (age-specific) incidence into high- and low-risk groups. This approach provided age-specific and risk-stratified incidence across hospital, ED, and PO settings, anchored in Mexico-specific hospitalization and comorbidity data and supplemented by international evidence where needed (Table S2).

### 2.4. Mortality

Age-specific mortality rates were estimated using general mortality inputs obtained from the National Institute of Statistics and Geography (INEGI) [29] combined with risk distribution based on data from a large national cohort study in Mexico [4] (Table S3). The CFR for RSV-associated in-hospital mortality (8.6 deaths per 100 hospitalizations for adults aged 65–74 years) was based on a global systematic review of RSV burden in older adults from developed settings [9]. Age- and risk-specific distributions were informed by the published literature [30] (Table S4). General population mortality allocated by calendar month was estimated using 2024 data from the National Institute of Statistics and Geography (INEGI) (Table S5) [29].

### 2.5. Vaccine Effectiveness and Strategy

In the base case, RSVpreF was modeled as a single-dose vaccination given at model entry to adults aged 60–99 years. Vaccine uptake was set at 58%, chosen to reflect recent influenza vaccination coverage among Mexican adults aged ≥60 years reported in ENSANUT 2023 [19] and was applied uniformly across age and risk groups. No booster doses were included in the base case.

Vaccine effectiveness (VE) was informed by the latest real-world effectiveness data and clinical trial efficacy estimates. VE against RSV-related hospitalizations and ED visits was derived from the Kaiser Permanente Southern California (KPSC) study, which evaluated RSVpreF effectiveness against RSV-related lower respiratory tract disease hospitalizations and ED encounters among adults aged 18–99 years over the 2023–2025 RSV seasons [17,18]. In line with these data, initial VE against RSV-related hospitalization or ED visits was set at 87.9%, extrapolated from season 1 KPSC results that reported 87.0% (95% CI: 37–97) effectiveness at a mean follow-up of 1.9 months [17,18]. Effectiveness was then assumed to decline linearly from 2 to 12 months post-vaccination, reaching 77.0% (95% CI: 53–89) at 12.2 months (season 2 estimate), with further linear waning thereafter such that protection persisted through month 90 and was assumed to be 0% from month 91 onward. This time-varying VE profile was applied to both hospitalization and ED outcomes.

VE against RSV disease in primary/outpatient (PO) settings was based on full season 1 and 2 results from the phase 3 RENOIR trial and associated post hoc analyses in adults aged ≥60 years [16]. Because RENOIR endpoints were defined by clinically confirmed RSV illness rather than by care setting, we used vaccine efficacy against medically attended RSV acute respiratory illness as a surrogate for VE against RSV-related PO visits [16].

### 2.6. Utilities and Disutilities

Age-specific health state utilities for older adults were obtained from a Mexico-specific source and applied to the modeled cohort by single year of age [31]. Baseline utilities were further stratified by risk status (high vs. low risk) using relative adjustments derived from the published literature in adult populations [32,33] (Table S6).

Temporary quality-of-life decrements associated with RSV illness were incorporated as disutilities. A QALY loss of 0.0167 was applied for RSV-related hospitalizations and 0.0054 for RSV-related ED/PO visits, based on estimates of the short-term health impact of RSV and influenza in older adults. These disutilities were applied in the cycle in which the RSV event occurred and combined with age- and risk-specific baseline utilities to estimate total QALYs over the model horizon [34,35].

### 2.7. Costs

Direct medical care costs were assigned to each RSV event type using Mexican public payer unit costs [36]. RSV-related hospitalization was assumed to be MXN$ 111,133 (US$ 6052) per episode. The unit cost for a hospital day (MXN$ 15,018) was derived from Mexican public payer unit costs [36] and then was multiplied by 7.4, the mean length of hospital stay in University Hospital “Dr. José Eleuterio González” during the winter season (October 2023–March 2024) [37]. RSV-related ED visits [MXN$ 3857 (US$ 210)] and RSV-related PO visits [MXN$ 1296 (US$ 71)] costs were estimated using Mexican public payer unit costs [36].

The vaccine price for RSVpreF was determined as a value-based/economically justifiable price (EJP), defined as the price at which RSVpreF vaccination is cost-effective compared with no vaccination. Specifically, the model solved for the price at which the incremental cost-effectiveness ratio (ICER) equals a willingness-to-pay threshold of MXN$ 259,159 (US$ 14,158) per QALY gained, corresponding to 1× Mexico’s 2024 gross domestic product per capita (GDPpc) [38,39,40].

Indirect costs related to productivity losses for patients and caregivers were estimated based on workforce participation rates, average daily wages, and the number of workdays lost due to RSV-related illness. For patients and caregivers, workforce participation rates (Table S7) and average daily wage (Table S8) stratified by age were obtained from the National Institute of Statistics and Geography (INEGI) [41,42]. The number of workdays lost (10.7 days) was derived from a published study estimating productivity losses based on respiratory disease-related short-term disability claims (RD-STDC) submitted in 2020 to the Mexican Social Security Institute (Instituto Mexicano del Seguro Social [IMSS]) among approximately 19.1 million insured workers [43].

All costs were expressed in 2025 Mexican pesos (MXN), with secondary presentation in US dollars (US$) using an exchange rate of MXN$ 18.4 per US$ obtained from Xe [44] and based on September 2025 exchange rate data.

### 2.8. Base-Case Analysis

The base-case analysis compared a single-dose RSVpreF vaccination with no vaccination in adults aged 60–99 years in Mexico over a lifetime time horizon. Results were generated from both the Mexican healthcare system perspective and a societal perspective. Consistent with the model structure, costs and health outcomes were discounted at 5% per year [45]. Model outputs included RSV clinical outcomes (medically attended cases by care setting, hospitalizations, and in-hospital deaths) and economic outcomes (total and incremental costs), summarized as LYs, QALYs, and the ICER for vaccination versus no vaccination. Cost-effectiveness was assessed using a benchmark threshold of 1 GDPpc per QALY gained for Mexico (MXN$ 259,159 [US$ 14,158]).

### 2.9. Sensitivity Analysis

To assess the robustness of the results, a one-way sensitivity analysis (OWSA) was performed. OWSA was performed by varying disease incidence, CFR due to RSV hospitalization, vaccine effectiveness, direct and indirect costs of disease, and utilities and disutilities for healthy adults by ±25%. This ±25% range was applied as a standardized assumption across inputs to reflect plausible parameter uncertainty where setting-specific uncertainty estimates were not consistently available from the literature.

A probabilistic sensitivity analysis (PSA) was also conducted to characterize joint uncertainty across model inputs. The PSA comprised 1000 Monte Carlo replications. In each replication, RSV disease rates for hospitalization, ED, and PO encounters; general population mortality; the CFR for RSV hospitalization; RSVpreF effectiveness against hospitalization, ED, and PO outcomes; healthy-adult utility; and acute-phase disutilities associated with hospitalization and ED/PO encounters were sampled from beta distributions. Direct medical costs per RSV-related hospitalization, ED visit, and PO visit were sampled from log-normal distributions parameterized using the specified means and standard errors. Parameters without an assigned probability distribution were held at their base-case values.

The PSA was evaluated from the healthcare-system perspective using the deterministic EJP as the fixed per-dose vaccine price in all replications. Because this price was estimated by setting the deterministic ICER equal to the willingness-to-pay threshold, the deterministic base-case result lay on the cost-effectiveness threshold by construction. The PSA therefore evaluated whether the deterministically estimated break-even vaccine price remained cost-effective after jointly accounting for parameter uncertainty.

Probabilistic results were summarized as mean incremental healthcare costs, QALYs, and ICERs. The corresponding 95% uncertainty intervals were reported for incremental healthcare costs and QALYs. Joint uncertainty in incremental costs and QALYs was presented on the cost-effectiveness plane. A cost-effectiveness acceptability curve (CEAC) was generated by calculating, at each willingness-to-pay threshold, the proportion of replications in which RSVpreF vaccination produced a non-negative incremental net monetary benefit.

### 2.10. Scenario Analysis

Scenario analyses were conducted to explore policy-relevant alternative assumptions that are not well captured by one-way parameter variation, and to complement the OWSA by testing discrete changes to the model inputs and target population while holding all other model features constant. In all scenarios, the vaccine price was fixed at the EJP derived in the base case from the healthcare system perspective; only the parameters described below were varied. For each scenario, the EJP required to meet the willingness-to-pay threshold of one GDPpc per QALY was recalculated and is reported in the final row of the scenario analysis table.

First, to assess the impact of restricting burden estimates to respiratory diagnoses only, an alternative epidemiological scenario using RSV-attributable respiratory-only hospitalization rates from Burkart et al. [8] instead of the base-case cardiorespiratory hospitalization rates was examined. This scenario reflects a more conservative approach that excludes RSV-attributable cardiorespiratory exacerbations and cardiovascular events, which have been increasingly recognized as part of the broader RSV burden in older adults and have been highlighted in recent evidence supporting RSVpreF effectiveness against cardiorespiratory outcomes [46,47,48].

Second, to explore the potential public health impact of prioritizing different population subgroups, alternative age- and risk-based vaccination strategies were evaluated: (1) vaccinating only high-risk adults aged 60–99 years; (2) vaccinating all adults aged 75–99 years (regardless of risk); and (3) vaccinating high-risk adults aged 60–74 years plus all adults aged 75–99 years.

Third, to evaluate the impact of effective coverage under different implementation conditions, vaccine uptake was varied to 40% and 80% compared to base-case uptake of 58%, while keeping the same target population as in the base case.

Fourth, to evaluate the impact of a shorter duration of vaccine protection, vaccine effectiveness against RSV-associated hospitalization and ED visits was modeled using a more conservative waning profile, with effectiveness declining linearly to 0% by 56 months [49].

Fifth, to address uncertainty in comorbidity distribution, which impacts the derivation of age-/risk-specific incidence rates, scenario analyses were conducted using alternative US-specific data sources [28].

Each scenario was run separately using the same model structure, discounting, and costing approach as the base case, and results were summarized as incremental clinical outcomes, costs, and ICERs for RSVpreF vaccination versus no vaccination.

## 3. Results

### 3.1. Base-Case Analysis

In the base case without vaccination, RSV was projected to impose a substantial lifetime burden on older adults in Mexico, with a total of 15,707,653 medically attended cases. (Table 1). Across the modeled cohort, RSV was estimated to cause 2,146,234 hospitalizations, 2,028,379 ED encounters, and 11,533,040 PO encounters.

Introducing RSVpreF vaccination with 58% uptake markedly reduced this burden. Compared with no vaccination, RSVpreF was projected to reduce RSV hospitalizations to 1,958,409 (a decrease of 187,825), ED encounters to 1,850,101 (a decrease of 178,278), and PO encounters to 11,067,064 (a decrease of 465,976). The model also estimated 15,384 fewer RSV-related deaths. In the first 5 years of the program alone, RSVpreF vaccination was projected to reduce RSV hospitalizations, ED encounters, and RSV-related deaths by 31% each, and PO encounters by 14%.

Over the full model time horizon (lifetime), these clinical benefits corresponded to gains of 96,227 discounted LYs and 71,526 discounted QALYs in the vaccinated cohort.

In terms of cost, vaccination generated substantial savings in RSV-related medical care. Direct medical care costs decreased from MXN$ 156,562 million (US$ 8526 million) in the no-vaccination scenario to MXN$ 137,077 million (US$ 7465 million) with RSVpreF, yielding savings of MXN$ 19,484 million (US$ 1061 million). These were offset by vaccination costs of MXN$ 38,021 million (US$ 2074 million), resulting in total direct costs of MXN$ 175,098 million (US$ 9539 million) with vaccination versus MXN$ 156,562 million (US$ 8526 million) without, resulting in an incremental MXN$ 18,537 million (US$ 1013 million).

When indirect costs were included, RSVpreF reduced productivity losses due to both morbidity and mortality. Total indirect costs decreased from MXN$ 29,518 million (US$ 1608 million) without vaccination to MXN$ 26,193 million (US$ 1426 million) with vaccination, corresponding to savings of MXN$ 3324 million (US$ 181 million). Total costs (direct + indirect) increased from MXN$ 186,079 million (US$ 10,134 million) in the no-vaccination scenario to MXN$ 201,291 million (US$ 10,965 million) with RSVpreF, a net increment of MXN$ 15,212 million (US$ 832 million).

These incremental costs and health gains yielded, from the healthcare system perspective, an ICER of MXN$ 192,634 (US$ 10,524) per LY gained, and MXN$ 259,159 (US$ 14,158) per QALY gained. From the societal perspective, after accounting for avoided productivity losses, the ICER improved to MXN$ 158,087 (US$ 8642) per LY gained and MXN$ 212,682 (US$ 11,627) per QALY gained.

The base-case analysis was parameterized to estimate the EJP from the healthcare system perspective, defined as the maximum vaccine price at which RSVpreF vaccination would remain cost-effective under the selected threshold and modeled assumptions. Under these conditions, the EJP was estimated at MXN$ 3709 (US$ 202) per dose from the healthcare system perspective and MXN$ 4034 (US$ 220) per dose from the societal perspective.

### 3.2. Sensitivity Analysis

**Figure 1:**
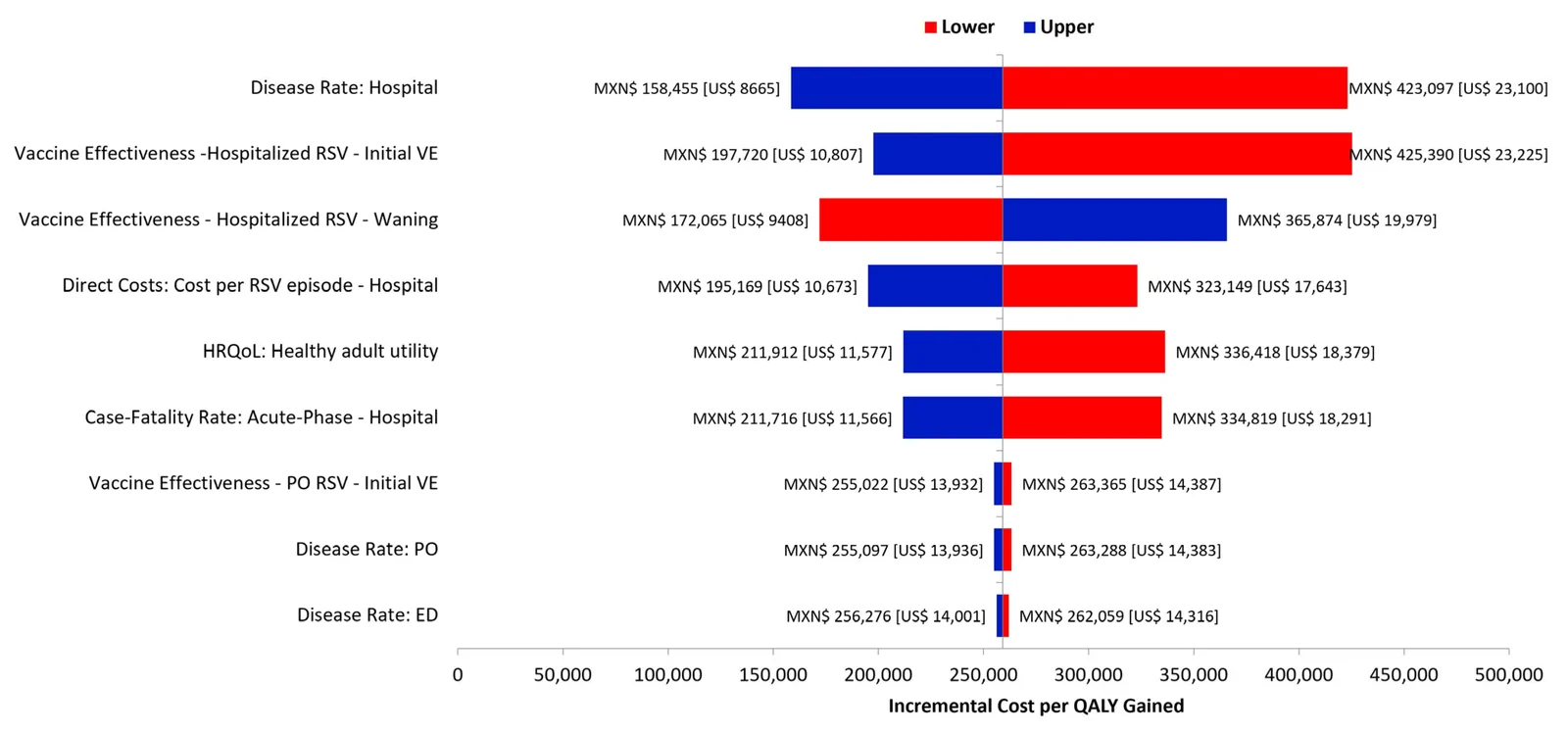
Tornado diagram for the one-way sensitivity analysis (upper [blue] and lower [red] bounds).

Results of the OWSA are presented as a tornado diagram (Figure 1), illustrating the impact of key model parameters on the ICER. Overall, the results indicate that the model outcomes were most sensitive to assumptions related to RSV disease burden and VE against severe outcomes.

The parameters with the greatest influence on the ICER were the RSV hospitalization rate, the initial VE against hospitalized RSV, and the waning of VE against hospitalized RSV. Variation in the RSV hospitalization rate resulted in ICERs ranging from MXN$ 158,455 (US$ 8665) to MXN$ 423,097 (US$ 23,100) per QALY gained from the healthcare system perspective. Similarly, uncertainty around initial VE and its waning over time led to substantial shifts in the ICER.

Other influential parameters included the direct cost per RSV-related hospitalization, baseline health-related quality of life (HRQoL) for healthy adults, and the CFR during the acute hospitalized phase. While variation in these parameters affected the ICER, their impact was more moderate compared with disease incidence and VE assumptions. In contrast, parameters related to RSV-PO events (e.g., PO disease rate and VE against non-hospitalized RSV) had relatively limited influence on the ICER.

Across all OWSAs, the direction and relative magnitude of effects were consistent with the base-case findings, indicating that the results are robust to plausible variations in key model inputs.

Across 1000 PSA replications, RSVpreF vaccination generated a mean of 77,221 additional discounted QALYs compared with no vaccination, with a 95% uncertainty interval of 11,250–203,075 QALYs. From the healthcare-system perspective, mean incremental healthcare costs were MXN$ 20,444 million (US$ 1111.1 million), with a 95% uncertainty interval of MXN$ 13,982–26,441 million (US$ 759.9–1437.0 million). The ICER based on mean probabilistic outcomes was MXN$ 264,758 (US$ 14,389) per QALY gained.

**Figure 2:**
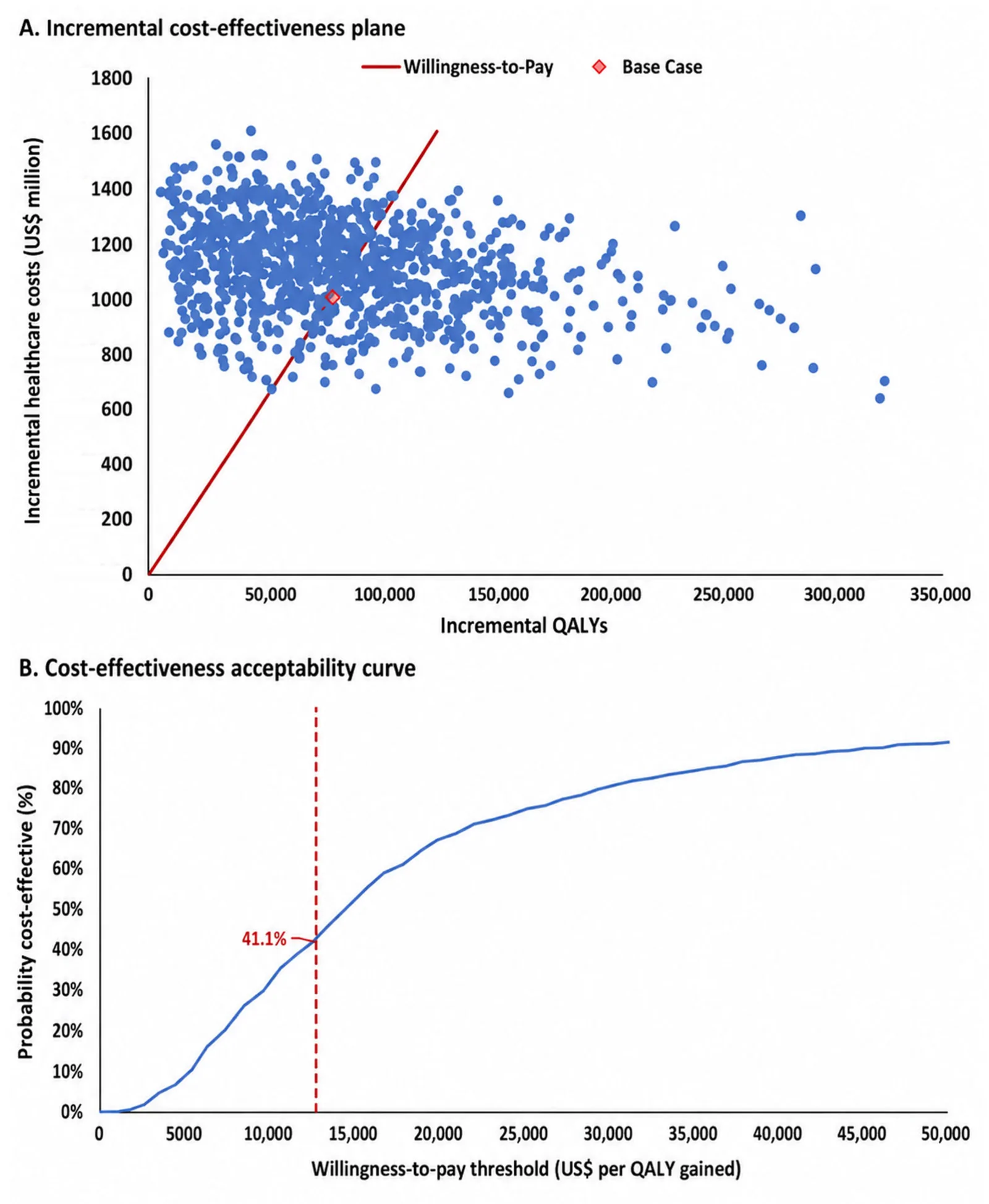
Probabilistic sensitivity analysis of RSVpreF vaccination versus no vaccination from the healthcare-system perspective. (**A**) Incremental cost-effectiveness plane showing 1000 probabilistic replications. The red line represents the willingness-to-pay threshold of MXN$ 259,159 (US$ 14,158) per QALY gained, and the red diamond represents the deterministic base-case result at the economically justifiable price. Replications below the line were considered cost-effective. (**B**) Cost-effectiveness acceptability curve showing the probability of cost-effectiveness across alternative willingness-to-pay thresholds; 41.1% of replications were cost-effective at the selected threshold.

On the cost-effectiveness plane, the deterministic base-case result lay on the willingness-to-pay line because the vaccine price was calibrated such that the deterministic ICER equaled the selected threshold. At the willingness-to-pay threshold of MXN$ 259,159 (US$ 14,158) per QALY gained, 41.1% of PSA replications produced a non-negative incremental net monetary benefit and were therefore considered cost-effective from the healthcare-system perspective (Figure 2).

### 3.3. Scenario Analysis

Incremental results for all scenario analyses are presented in Table 2. Across scenarios, RSVpreF vaccination remained clinically beneficial, with the magnitude of averted events varying by assumptions on RSV-attributable burden, target population, and uptake.

When burden estimates were restricted to RSV-attributable respiratory hospitalization outcomes only (Scenario 1), the projected health impact of vaccination was smaller than in the base case, which captures the broader cardiorespiratory hospitalization burden. Under the respiratory-only scenario, RSVpreF was projected to avert 99,235 hospitalizations and 8160 deaths, yielding 38,192 QALYs gained, compared with 187,825 hospitalizations and 15,384 deaths averted and 71,526 QALYs gained in the base case. Consistent with the lower burden captured under respiratory-only outcomes, the economic results were less favorable than in the base case.

Across subgroup strategies, narrowing eligibility reduced the number of people vaccinated and the absolute number of RSV events averted compared with the base case (vaccinating all adults aged 60–99 years). The most restrictive strategy, i.e., vaccinating all adults aged 75–99 years, protected the smallest population (2,555,528 vaccinated) and yielded the smallest absolute benefit, averting 252,355 medically attended cases, 70,787 hospitalizations, and 7097 deaths. In contrast, the two broader targeting strategies than scenario 3, vaccinating high-risk adults aged 60–99 years (5,785,953 vaccinated) or high-risk adults aged 60–74 years plus all adults aged ≥75 years (6,738,879 vaccinated), averted a similar number of medically attended cases (636,332 and 681,189, respectively) and prevented 14,404–14,920 deaths, but still fell short of the overall impact achieved in the base case where all adults aged 60+ were eligible. From an economic standpoint, all subgroup strategies remained cost-effective, with healthcare-perspective ICERs of MXN$ 48,889 [US$ 2689], MXN$ 87,551 [US$ 4802], and MXN$ 90,216 [US$ 4943] per QALY for the high-risk 60–99, 75–99, and mixed (high-risk 60–74 + all ≥75) strategies, respectively, each well below the base-case ICER.

Varying uptake to 40% or 80% primarily scaled health benefits and cost offsets, with larger (smaller) reductions in RSV events and greater (smaller) QALY gains under higher (lower) uptake (e.g., 49,585 QALYs at 40% vs. 99,169 QALYs at 80%). Reducing vaccine uptake to 40% and increasing it to 80% proportionally changed the number of QALYs gained and medical care cost savings, while leaving the ICER unchanged because the incremental costs and outcomes scaled proportionally with coverage.

Assuming a more conservative duration of protection, with vaccine effectiveness waning to 0% by 56 months, reduced QALYs gained (+38,870 vs. 71,526 base case) and increased the ICER to MXN$ 707,136 (US$ 38,592) [49].

Under alternative burden assumptions, applying US-specific risk prevalence from Weycker et al. [28] slightly decreased the number of medically attended cases prevented (to −185,014 hospitalizations; −15,362 deaths) and increased the healthcare perspective ICER to MXN$ 260,478 (US$ 14,229).

## 4. Discussion

To the best of our knowledge, this is the first economic evaluation of RSV vaccination in older adults in Mexico. Our findings suggest that a single dose of RSVpreF administered year-round is likely to represent good value for money in Mexico and could generate meaningful public health benefits. At 58% uptake, the program would protect approximately 10.4 million adults aged ≥60 years and prevent a substantial vaccine-preventable burden, including more than 15,000 deaths and large numbers of medically attended RSV events over the cohort lifetime, while generating sizeable gains in discounted QALYs and reducing direct medical and productivity-related costs.

From an economic interpretation standpoint, a key policy-relevant output of this analysis is the value-based (economically justifiable) price implied by Mexico’s cost-effectiveness benchmark. Under a willingness-to-pay threshold of approximately 1× GDPpc per QALY gained, RSVpreF vaccination remains cost-effective up to an EJP of approximately MXN$ 4034 (US$ 220) per dose (societal perspective), with a corresponding base-case EJP of approximately MXN$ 3709 (US$ 202) per dose when calibrated to the healthcare system perspective. This approach is intended to assist results interpretation, illustrating the maximum vaccine price at which the program would remain cost-effective under Mexico’s benchmark (i.e., the EJP). This provides a more informative policy perspective than an analysis based on an assumed price alone, as it identifies the price ceiling compatible with cost-effectiveness under Mexico’s threshold.

The PSA findings should be interpreted in the context of the value-based pricing framework used in this analysis. The vaccine price was calibrated so that the deterministic healthcare-system ICER equaled the selected willingness-to-pay threshold; consequently, the deterministic base-case result lay on the threshold by construction. The PSA therefore assessed uncertainty around whether this deterministically estimated break-even price would remain cost-effective when uncertainty in the model inputs was considered jointly. Under this framework, a probability of cost-effectiveness near one-half may be expected, although an exact probability of 50% is not required because the joint distribution of incremental costs and QALYs may be asymmetric and is influenced by the variance and correlation of the sampled parameters [50]. In this analysis, 41.1% of replications were cost-effective at the selected threshold, while the ICER based on mean probabilistic outcomes remained close to the threshold. Similar findings have been reported in previous EJP-based analyses. For example, Zeevat et al. reported probabilities of cost-effectiveness of 44% in the Netherlands and 59% in the United Kingdom at the willingness-to-pay thresholds used to estimate the corresponding RSV vaccine prices [51].

These findings are broadly consistent with evidence from other settings indicating that RSV in older adults is associated with high healthcare utilization and meaningful economic burden, and that preventing severe RSV outcomes through vaccination can provide substantial health and economic value [10,51,52,53,54]. Although direct comparisons across countries are limited by differences in epidemiology, healthcare costs, and decision thresholds, our results align qualitatively with prior evaluations.

Scenario analyses reinforce several policy-relevant insights. First, the base-case findings, grounded in RSV-attributable cardiorespiratory hospitalization outcomes, suggest that the public health value of vaccination may be greater when the broader severe burden of RSV in older adults is captured beyond respiratory diagnoses alone. This is particularly relevant in Mexico, where approximately 18.3 million people are aged 60+ and the prevalence of chronic comorbidities is high (~60%), increasing vulnerability to severe cardiorespiratory exacerbations. Under-ascertainment remains a major public health challenge, as routine RSV testing in adults is infrequent and symptoms overlap with other respiratory infections, so recent work that aims to better quantify the underlying burden is particularly relevant [5,6,7]. In this context, RSVpreF vaccination could be a meaningful public health tool to help reduce pressure on an already overburdened health system by preventing a substantial, increasingly vaccine-preventable burden of severe RSV-associated outcomes. Consistent with this interpretation, the respiratory-only scenario (Scenario 1) produced smaller estimated health gains and less favorable economic results, highlighting that restricting burden estimates to respiratory outcomes alone may understate the full clinical and economic impact of RSV in older adults [46,47,48].

Second, subgroup scenarios highlight important trade-offs between prioritization and equity. Targeting vaccination to high-risk adults aged 60–99 years is likely the most cost-effective approach, while vaccinating adults aged ≥75 years remains highly cost-effective. However, these strategies would leave a large risk group of older adults unprotected (8,446,744 adults aged 60+ without other risk factors and/or 13,783,545 adults aged 60–74 years). Because vaccinating all adults aged ≥60 years remains cost-effective under Mexico’s benchmark, a universal age-based strategy may represent an equitable and efficient use of public resources by offering protection across the broader older adult population rather than restricting access only to narrowly defined risk groups.

Third, uptake assumptions underscore the importance of implementation for realizing program impact. While cost-effectiveness results are stable under alternative uptake assumptions in this analysis, higher uptake translates into substantially larger population health benefits and greater reductions in medically attended cases. Moving from 40% to 80% uptake effectively doubles the number of vaccinated individuals and yields larger reductions in RSV-related events (7,241,939 increase in the number of people protected and 576,832 increase in the number of medically attended cases prevented under 80% vs. 40%). These results emphasize that achieving high coverage through delivery infrastructure, provider engagement, and public confidence will be critical to maximizing the public health value of RSV vaccination in Mexico.

Fourth, the scenario assuming a more conservative duration of vaccine protection highlights the importance of long-term vaccine effectiveness in determining the value of RSV vaccination. As expected, more rapid waning reduced the projected health benefits and resulted in less favorable cost-effectiveness outcomes. The more modest health gains observed under the conservative waning scenario likely reflect the use of VE estimates based on a less severe outcome definition (RSV-ARI), whereas the base case relied on VE data aligned with more severe outcomes (RSV-LRTD), such as hospitalization and death—consistent with the endpoints modeled in this analysis.

Fifth, the analysis was also robust to alternative assumptions regarding the distribution of high- and low-risk older adults. Applying risk stratification estimates derived from a US population had only a modest impact on the projected health and economic outcomes, with conclusions remaining consistent with those of the base-case analysis. This finding suggests that the model results are not driven by the specific approach used to stratify the population by risk, providing additional confidence in the overall robustness of the analysis despite the current lack of Mexico-specific data describing RSV risk by comorbidity status.

The analysis is subject to several acknowledged limitations. We assumed uniform costs across age and high- and low-risk groups in the absence of detailed local estimates. This analysis did not include several outcomes of RSV burden that would increase the estimated value of vaccination. Specifically, we did not model non–medically attended RSV cases, short- and longer-term sequelae of severe RSV (e.g., post-discharge care needs, frailty, and longer-term functional decline), or broader system-level effects (e.g., hospital capacity freed by prevented admissions and reduced antibiotic use). Additionally, the use of a static modeling approach does not capture indirect (herd immunity) effects. As a result, potential reductions in transmission to other high-risk groups, including other adults, are not reflected in the analysis, which may underestimate the overall public health impact. These conservative assumptions mean our estimates of cost-effectiveness may be lower-bound estimates of the true value. Conversely, some modeling assumptions may result in overestimation of the burden and subsequent benefits of vaccination. These include the attribution of RSV-associated cardiorespiratory hospitalizations and the extrapolation of vaccine effectiveness beyond the available follow-up period. To assess the impact of these assumptions, we evaluated scenario analyses using respiratory outcomes only and more conservative vaccine effectiveness assumptions. In addition, due to limited Mexico-specific data, several key epidemiologic inputs were informed using external evidence. Specifically, ED and PO incidence estimates were sourced from Li et al. [27], and relative risks used to stratify incidence by comorbidity profile across settings were derived from Weycker et al. [28]; similarly, RSV hospitalization CFR was drawn from a global systematic review based largely on developed settings [9]. These assumptions may introduce uncertainty if patterns of healthcare utilization or risk gradients differ in Mexico. Additionally, booster-dose scenarios were not evaluated due to the lack of clinical data to parameterize revaccination in older adults. Also, the use of a 1× GDPpc threshold estimate (MXN$ 259,159 [US$ 14,158]) as a proxy for the cost-effectiveness threshold has been criticized for its limited theoretical validity and potentially leading to inefficient resource allocation decisions that may reduce rather than improve overall population health [55]. Nonetheless, Mexico does not have an explicit cost-effectiveness threshold, and the lower bound of the WHO CHOICE recommendation [56] remains a practical reference, consistent with previously published economic evaluations for Mexico and across geographies [57,58,59]. Finally, VE estimates were derived from U.S.-based real-world effectiveness data due to the absence of local data; although informative, these findings may not fully reflect effectiveness in the Mexican population, and future analyses should incorporate country-specific estimates as they become available. Despite these limitations, the robustness of results across scenarios and the consistency with external data suggest RSVpreF vaccination in older adults in Mexico would provide substantial health and economic benefits.

## 5. Conclusions

Year-round RSVpreF vaccination among older adults in Mexico is projected to substantially reduce RSV-related hospitalizations, mortality, and associated healthcare utilization, while remaining cost-effective under the selected benchmark. In the context of Mexico’s rapidly aging population and the broader RSV-attributable burden, these findings underscore the importance of preventive strategies that address the growing clinical and economic impact of RSV in older adults. Incorporating RSVpreF vaccination into the national immunization program may help optimize hospital resource use and improve health outcomes among older adults.

## Figures and Tables

**Table 1:** Base-case analysis results.

	No Vaccination	RSVpreF Vaccination	∆
Eligible population aged 60+	18,347,430	18,347,430	0
Number of people vaccinated, assuming 58% uptake	0	10,446,497	10,446,497
Clinical outcomes (events)			
RSV hospitalizations	2,146,234	1,958,409	−187,825
RSV ED encounters	2,028,379	1,850,101	−178,278
RSV PO encounters	11,533,040	11,067,064	−465,976
Total No. of medically attended cases	15,707,653	14,875,574	−832,079
No. of RSV-related deaths	199,407	184,023	−15,384
Life years (discounted)	179,036,475	179,132,702	96,227
QALYs (discounted)	140,844,079	140,915,605	71,526
Economic outcomes (MXN$M [US$M])			
Direct			
Medical care	156,562 (8526)	137,077 (7465)	−19,484 (−1061)
Vaccination	0	38,021 (2074)	38,021 (2074)
Total direct	156,562 (8526)	175,098 (9539)	18,537 (1013)
Indirect			
Morbidity-related productivity loss	22,223 (1210)	20,258 (1103)	−1965 (−107)
Mortality-related productivity loss	7294 (397)	5935 (323)	−1359 (−74)
Total indirect	29,518 (1608)	26,193 (1426)	−3324 (−181)
Direct + indirect	186,079 (10,134)	201,291 (10,965)	15,212 (832)
ICER (MXN$ [US$]) Healthcare Perspective			
Cost per LY			192,634 (10,524)
Cost per QALY			259,159 (14,158)
ICER (MXN$ [US$]) Societal Perspective			
Cost per LY			158,087 (8642)
Cost per QALY			212,682 (11,627)

MXN$ = Mexican Pesos, ED = emergency department, ICER = incremental cost-effectiveness ratio, LY = life year, M = million, PO = physician office, QALY = quality-adjusted life year, RSV = respiratory syncytial virus, US$ = United States Dollar.

**Table 2:** Scenario analysis results: difference between No vaccination and RSVpreF.

	Base Case *	Scenario 1 RSV Respiratory Outcomes [8]	Scenario 2 60–99 (High Risk)	Scenario 3 75–99 Years	Scenario 4 60–74 (High Risk) and 75–99 Years	Scenario 5 40% Uptake	Scenario 6 80% Uptake	Scenario 7 Conservative Vaccine Effectiveness [49]	Scenario 8 United States Risk Stratification [28]
Number of people vaccinated	10,446,497	10,447,932	5,785,953	2,555,528	6,738,879	7,241,939	14,483,878	10,446,497	10,446,966
Clinical outcomes (events)									
RSV hospitalizations	−187,825	−99,235	−174,091	−70,787	−179,197	−130,208	−260,416	−94,626	−185,014
RSV ED encounters	−178,278	−179,356	−135,987	−55,544	−146,869	−123,589	−247,179	−97,504	−176,765
RSV PO encounters	−465,976	−471,114	−326,255	−126,025	−355,123	−323,034	−646,068	−471,372	−464,599
Total No. of medically attended cases	−832,079	−749,705	−636,332	−252,355	−681,189	−576,831	−1,153,663	−663,502	−826,379
No. of RSV-related deaths	−15,384	−8160	−14,404	−7097	−14,920	−10,665	−21,330	−7660	−15,362
Life years (discounted)	96,227	49,309	89,980	31,659	92,408	66,708	133,417	50,708	97,473
QALYs (discounted)	71,526	38,192	65,390	22,913	67,652	49,585	99,169	38,870	72,203
Economic outcomes (MXN$M [US$M])									
Direct									
Medical care	−19,484 (−1061)	−10,832 (−590)	−17,862 (−973)	−7296 (−397)	−18,424 (−1003)	−13,507 (−736)	−27,015 (−1471)	−10,535 (−574)	−19,215 (−1046)
Vaccination	38,021 (2074)	38,026 (2074)	21,059 (1149)	9302 (507)	24,527 (1338)	26,358 (1438)	52,716 (2875)	38,021 (2074)	38,023 (2074)
Total direct	18,537 (1013)	27,195 (1484)	3197 (176)	2006 (110)	6103 (334)	12,850 (702)	25,701 (1404)	27,486 (1500)	18,807 (1027)
Indirect									
Morbidity-related productivity loss	−1965 (−107)	−1782 (−97)	−1474 (−80)	−417 (−23)	−1549 (−84)	−1362 (−74)	−2725 (−148)	−1617 (−88)	−1953 (−106)
Mortality-related productivity loss	−1359 (−74)	−654.21 (−36)	−1283 (−70)	−136 (−7)	−1291 (−70)	−942 (−51)	−1884 (−103)	−747 (−41)	−1359 (−74)
Total indirect	−3324 (−181)	−2436 (−133)	−2758 (−150)	−552 (−30)	−2840 (−155)	−2305 (−126)	−4609 (−251)	−2365 (−129)	−3312 (−180)
Direct + indirect	15,212 (832)	24,759 (1352)	439 (26)	1454 (80)	3263 (180)	10,546 (577)	21,092 (1153)	25,122 (1371)	15,495 (847)
ICER (MXN$ [US$]) healthcare perspective									
Cost per LY	192,634 (10,524)	551,512 (30,099)	35,529 (1954)	63,365 (3475)	66,047 (3619)	192,634 (10,524)	192,634 (10,524)	542,046 (29,582)	192,950 (10,540)
Cost per QALY	259,159 (14,158)	712,056 (38,861)	48,889 (2689)	87,551 (4802)	90,216 (4943)	259,159 (14,158)	259,159 (14,158)	707,136 (38,592)	260,478 (14,229)
ICER (MXN$ [US$]) societal perspective									
Cost per LY	158,087 (8642)	502,110 (27,409)	4879 (285)	45,924 (2525)	35,314 (1945)	158,087 (8642)	158,087 (8642)	495,416 (27,043)	158,967 (8690)
Cost per QALY	212,682 (11,627)	648,273 (35,388)	6714 (393)	63,453 (3489)	48,236 (2657)	212,682 (11,627)	212,682 (11,627)	646,303 (35,279)	214,601 (11,731)
EJP for each scenario (MXN$ [US$])	3709 (202)	2022 (110)	6131 (334)	5278 (288)	5438 (297)	3709 (202)	3709 (202)	2011 (110)	3700 (202)

* at assumed 58% uptake. MXN$ = Mexican Pesos, US$ = United States Dollar, ED = emergency department, ICER = incremental cost-effectiveness ratio, LY = life year, M = million, PO = physician office, QALY = quality-adjusted life year, RSV = respiratory syncytial virus.

## Data Availability

The data presented in this study are available upon request from the corresponding author.

## Supplementary Materials

The following supporting information can be downloaded at https://www.mdpi.com/article/10.3390/vaccines14080713/s1. Figure S1. Model diagram. Table S1. Risk distribution, by age (years). Table S2. Age- and risk-specific rates of RSV encounters (per 100,000). Table S3. Age- and risk-specific general population mortality rates. Table S4. Age- and risk-specific hospital case fatality rates (per 100). Table S5. Distribution of general population mortality by calendar month. Table S6. Age- and risk-specific general population health state utility. Table S7. Age-specific workforce participation. Table S8. Age-specific average daily wage.
