# Supplementary material for: Cost-Effectiveness Analysis of Older Adult Vaccination with the Bivalent Respiratory Syncytial Virus Prefusion F (RSVpreF) Vaccine in Mexico
Source: Vaccines (Basel). 2026 Aug 19;14(8):713. doi: 10.3390/vaccines14080713 (PMC13517743; doi:10.3390/vaccines14080713)
Supplement: Supplementary file 1 [file vaccines-14-00713-s001.zip › vaccines-4401190-supplementary.pdf]

vaccines MDPI

Supplementary Materials

Figure S1. Model diagram

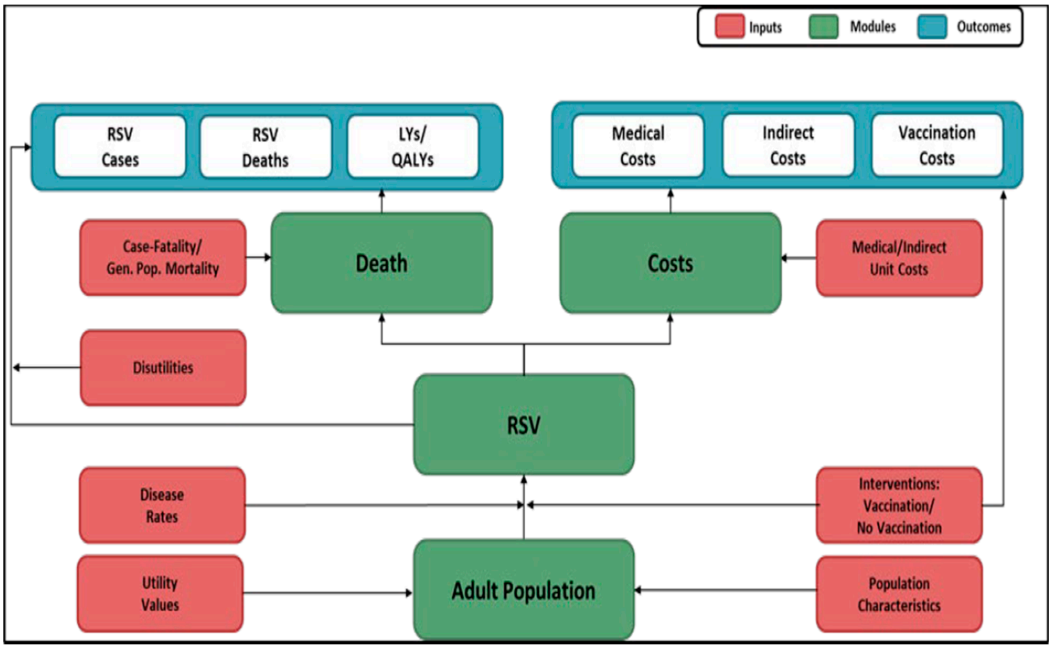

LYs: life years; QALYs: quality adjusted life years; RSV: respiratory syncytial virus

Notes: The model uses a monthly cycle length; Vaccine effectiveness wanes over time and is applied dynamically across cycles and the population is stratified by age and risk group, with inputs and outcomes estimated separately for each subgroup

Table S1. Risk distribution, by age (years) [1].

Age	Low risk	High risk
18-49 years	78.6%	21.4%
50-64 years	55.9%	44.1%
65-74 years	42.7%	57.3%
75-84 years	38.0%	62.0%
85-99 years	33.5%	66.5%

High-risk individuals were defined as those with at least one of the following comorbidities: obesity, diabetes, hypertension, cardiovascular disease (CVD), chronic kidney disease (CKD), chronic obstructive pulmonary disease (COPD), immunosuppression (IS), asthma, smoking, or other comorbidity(ies). Low-risk individuals were defined as those without any of the listed conditions.

Table S2. Age- and risk-specific rates of RSV encounters (per 100,000) [1-3].

Age/Risk	Hospital	ED	PO
18-49 years			
Low risk	11.2	216.3	1870.5
High risk	201.2	649.0	3554.0
50-64 years			
Low risk	32.7	205.5	2405.4

High risk	546.5	513.8	4089.2
65-74 years			
Low risk	44.3	284.2	2421.2
High risk	811.2	795.8	4842.5
75-84 years			
Low risk	90.0	365.1	2589.3
High risk	1394.7	1058.9	5437.4
85-99 years			
Low risk	196.2	464.4	2548.2
High risk	1903.2	1300.3	6115.7

Low-risk and high-risk groups were defined in Table S1, ED = emergency department, PO = physician office, RSV = respiratory syncytial virus.

Table S3. Age- and risk-specific general population mortality rates [1,4].

Age	Low risk	High risk
18-49 years	0.2	0.3
50-64 years	0.7	1.2
65-74 years	1.6	2.8
75-84 years	3.5	6.1
85-99 years	8.9	15.6

Low-risk and high-risk groups were defined in Table S1.

Table S4. Age- and risk-specific hospital case fatality rates (per 100) [5,6].

Age	Low risk	High risk
18-49 years	0.2	1.5
50-64 years	2.1	5.1
65-74 years	3.9	7.0
75-84 years	7.5	9.5
85-99 years	11.8	10.6

Low-risk and high-risk groups were defined in Table S1.

Table S5. Distribution of general population mortality by calendar month [4].

Month	Percentage
January	9.5%
February	8.3%
March	8.2%
April	7.9%
May	9.1%
June	8.0%
July	8.0%
August	8.0%
September	7.7%
October	8.2%
November	8.0%
December	9.0%

Table S6. Age- and risk-specific general population health state utility [7,8].

Age	Low risk	High risk
18-49 years	0.929	0.733
50-64 years	0.920	0.694
65-74 years	0.913	0.707
75-84 years	0.907	0.682
85-99 years	0.901	0.643

Table S7. Age-specific workforce participation [9].

Age	Labour force participation (%)
18-49 years	70.7%
50-64 years	63.4%
65-74 years	34.5%
75-84 years	15.1%
85-99 years	0.6%

Table S8. Age-specific average daily wage [10].

Age	Average daily wage
18-49 years	MXN 405.26 (US$ 22.07)
50-64 years	MXN 415.76 (US$ 22.64)
65-74 years	MXN 337.97 (US$ 18.41)
75-84 years	MXN 337.97 (US$ 18.41)
85-99 years	MXN 337.97 (US$ 18.41)

1. Domínguez-Ramírez, L.; Sosa-Jurado, F.; Díaz-Sampayo, G.; Solis-Tejeda, I.; Rodríguez-Pérez, F.; Pelayo, R.; Santos-López, G.; Cortes-Hernandez, P. Age and comorbidities as risk factors for severe covid-19 in mexico, before, during and after massive vaccination. *Vaccines* **2023**, *11*.
2. Burkart, K.; Liang, C.; Rafferty, Q.; Gillespie, C.W.; McLaughlin, S.; Oros, A.; Suba, J.; Bruno, D.; Fahey, M.; Grajales, A.G., *et al.* Respiratory syncytial virus-attributable hospitalizations among adults in high- and middle-income countries: Application of the global burden of disease framework. *eClinicalMedicine* **2025**, *85*.
3. Weycker, D.; Averin, A.; Houde, L.; Ottino, K.; Shea, K.; Sato, R.; Gessner, B.D.; Yacisin, K.; Curcio, D.; Begier, E., *et al.* Rates of lower respiratory tract illness in us adults by age and comorbidity profile. *Infectious diseases and therapy* **2024**, *13*, 207-220.
4. National Institute of Statistics and Geography [Instituto Nacional de Estadística y Geografía]. General mortality [mortalidad general]. https://www.inegi.org.mx/sistemas/olap/registros/vitales/mortalidad/tabulados/def03.asp?t=02&c=11805 (20 October 2025),
5. Averin, A.; Shaff, M.; Weycker, D.; Lonshteyn, A.; Sato, R.; Pelton, S.I. Mortality and readmission in the year following hospitalization for pneumonia among us adults. *Respiratory medicine* **2021**, *185*, 106476.
6. Nguyen-Van-Tam, J.S.; O'Leary, M.; Martin, E.T.; Heijnen, E.; Callendret, B.; Fleischhackl, R.; Comeaux, C.; Tran, T.M.P.; Weber, K. Burden of respiratory syncytial virus infection in older and high-risk adults: A systematic review and meta-analysis of the evidence from developed countries. *European respiratory review : an official journal of the European Respiratory Society* **2022**, *31*.
7. Ara, R.; Brazier, J.E. Using health state utility values from the general population to approximate baselines in decision analytic models when condition-specific data are not available. *Value in health : the journal of the International Society for Pharmacoeconomics and Outcomes Research* **2011**, *14*, 539-545.

8. Mendes, D.; Averin, A.; Atwood, M.; Sato, R.; Vyse, A.; Campling, J.; Weycker, D.; Slack, M.; Ellsbury, G.; Mugwagwa, T. Cost-effectiveness of using a 20-valent pneumococcal conjugate vaccine to directly protect adults in england at elevated risk of pneumococcal disease. *Expert review of pharmacoeconomics & outcomes research* **2022**, *22*, 1285-1295.
9. National Institute of Statistics and Geography [Instituto Nacional de Estadística y Geografía]. National occupation and employment survey, population aged 15 years and over [encuesta nacional de ocupación y empleo (enoe), población de 15 años y más de edad]. https://en.www.inegi.org.mx/programas/enoe/15ymas/#tabular_data (20 October 2025),
10. National Institute of Statistics and Geography [Instituto Nacional de Estadística y Geografía]. National household income and expenditure survey. 2024 new series [encuesta nacional de ingresos y gastos de los hogares (enigh). 2024 nueva serie]. https://www.inegi.org.mx/contenidos/saladeprensa/boletines/2025/enigh/ENIGH2024_RR.pdf (20 October 2025),

Disclaimer/Publisher's Note: The statements, opinions and data contained in all publications are solely those of the individual author(s) and contributor(s) and not of MDPI and/or the editor(s). MDPI and/or the editor(s) disclaim responsibility for any injury to people or property resulting from any ideas, methods, instructions or products referred to in the content.
